# Growth-Promoting Effect of Rhizobacterium (*Bacillus subtilis* IB22) in Salt-Stressed Barley Depends on Abscisic Acid Accumulation in the Roots

**DOI:** 10.3390/ijms221910680

**Published:** 2021-10-01

**Authors:** Zarina Akhtyamova, Tatiana Arkhipova, Elena Martynenko, Tatyana Nuzhnaya, Ludmila Kuzmina, Guzel Kudoyarova, Dmitry Veselov

**Affiliations:** 1 Ufa Institute of Biology, Ufa Federal Research Center, Russian Academy of Sciences, 450054 Ufa, Russia; akhtyamovazarina@gmail.com (Z.A.); tnarkhipova@mail.ru (T.A.); evmart08@mail.ru (E.M.); tanyawww89@mail.ru (T.N.); ljkuz@anrb.ru (L.K.); guzel@anrb.ru (G.K.); 2 Institute of Biochemistry and Genetics, Ufa Federal Research Center, Russian Academy of Sciences, 450054 Ufa, Russia

**Keywords:** *Hordeum vulgare* L., salt stress, abscisic acid, ABA-deficient mutant Az34, *Bacillus subtilis*

## Abstract

An ABA-deficient barley mutant (Az34) and its parental cultivar (Steptoe) were compared. Plants of salt-stressed Az34 (100 mmol m^−3^ NaCl for 10 days) grown in sand were 40% smaller than those of “Steptoe”, exhibited a lower leaf relative water content and lower ABA concentrations. Rhizosphere inoculation with IB22 increased plant growth of both genotypes. IB22 inoculation raised ABA in roots of salt-stressed plants by supplying ABA exogenously and by up-regulating ABA synthesis gene *HvNCED2* and down-regulating ABA catabolic gene *HvCYP707A1*. Inoculation partially compensated for the inherent ABA deficiency of the mutant. Transcript abundance of *HvNCED2* and related *HvNCED1* in the absence of inoculation was 10 times higher in roots than in shoots of both mutant and parent, indicating that ABA was mainly synthesized in roots. Under salt stress, accumulation of ABA in the roots of bacteria-treated plants was accompanied by a decline in shoot ABA suggesting bacterial inhibition of ABA transport from roots to shoots. ABA accumulation in the roots of bacteria-treated Az34 was accompanied by increased leaf hydration, the probable outcome of increased root hydraulic conductance. Thereby, we tested the hypothesis that the ability of rhizobacterium (*Bacillus subtilis* IB22) to modify responses of plants to salt stress depends on abscisic acid (ABA) accumulating in roots.

## 1. Introduction

High concentrations of ions in soil solution inhibit plant growth and decrease their productivity due to reduced availability of water and toxicity of ions, NaCl being the principal cause of soil salinity stress. Plant growth-promoting rhizobacteria (PGPR) increases the ability of plants to cope with salt stress [1,2,3,4]. However, mechanisms enabling this effect are still not clear enough, while their elucidation may contribute to a better understanding of how to increase plant salt tolerance in general. Among other mechanisms, the capacity of PGPR to produce plant hormones and to influence hormonal concentration in planta is believed to be involved not only in plant growth promotion but also in bacterial impact on plant stress resistance [5,6,7,8]. In this regard, most attention is paid to such hormones as cytokinins and auxins [9,10,11,12,13]. Abscisic acid (ABA) was less studied, although it is the latter hormone that is most involved in plants’ stress responses [14]. Nevertheless, there are reports showing the ability of bacteria either to produce [15] or catabolize ABA [16,17] and influence the concentration of this hormone in plants [18,19]. Production of ABA by plants has been shown to be important for plant response to PGPR [20]: unlike wild-type tomato plants, in which PGPR stimulated growth, bacteria inhibited the growth of ABA-deficient mutant plants. However, ABA-deficient monocot plants have not been used to study the dependence of plant response to PGPRs on ABA production by plants. In accordance, the goal of the present research was to reveal this dependence (or its absence) by comparing the effects of PGPRs on the growth and hormone content in salt-stressed ABA-deficient barley mutant Az34 and its parental cultivar Steptoe. Since PGPRs have been shown to influences ABA metabolism in planta [20,21], the expression of the genes involved in this process was analyzed. We studied the expression of the barley genes described previously [22]: *HvNCED1* and *HvNCED2* genes encoding dioxygenase of 9-cis-epocorotinoids (key enzyme for ABA synthesis) and *HvCYP707A1* gene-encoding ABA 8′-hydroxylase involved in ABA catabolism. The ability of the bacterial strain to synthesize or catabolize ABA was also studied to check whether PGPRs could have a direct effect on ABA content.

## 2. Results

**Figure 1:**
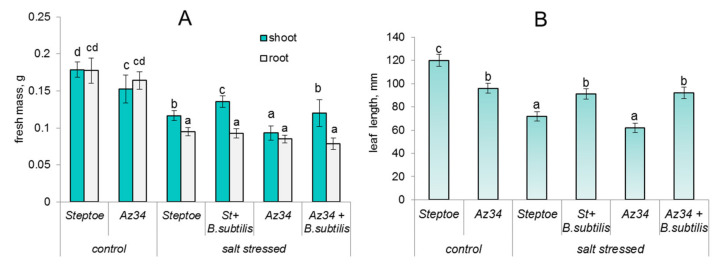
Influence of *B. subtilis* IB-22 on the fresh weights of shoots and roots (**A**) and leaf length (**B**) of control (unstressed) and salt-stressed (100 mM NaCl) ABA-deficient barley mutant (Az34) and its parental cultivar Steptoe measured on the 8th day after bacterial treatment. Significantly different means for each variable are labeled with different letters at *p* ≤ 0.05, *n* = 30.

In the absence of salt stress, the shoot mass of Az34 plants was about 15% lower than that of Steptoe, while root mass was similar in the plants of both genotypes (Figure 1A).

Salt stress inhibited plant growth manifested in decline in the mass of both shoots and root of either genotype. Introduction of *Bacillus subtilis* IB-22 into the rhizosphere increased shoot mass of either salt-stressed Az34 or Steptoe, while their root mass was not influenced by bacterial treatment.

Measuring the length of the second leaf showed that leaves of Az34 were smaller than of Steptoe either under normal or stress conditions; their size was decreased by salinity and increased by bacterial treatment (Figure 1B).

**Figure 2:**
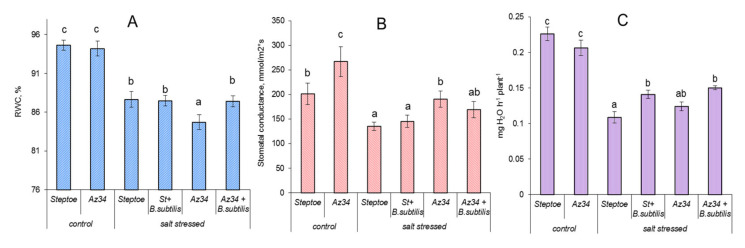
Influence of *B. subtilis* IB-22 on the relative water content in the leaves (**A**, *n* = 5) stomatal conductance (**B**, *n* = 40) and transpiration (**C**, *n* = 5) of control (unstressed) and salt-stressed (100 mM NaCl) ABA-deficient barley mutant (Az34) and its parental cultivar Steptoe measured on the 8th day after bacterial treatment. Significantly different means for each variable are labeled with different letters at *p* ≤ 0.05.

Relative water content (RWC) in the leaves of both genotypes was about 94% in the absence of salt stress (Figure 2A). The addition of NaCl to the nutrient solution resulted in about a 6% decline in RWC in almost all cases, except Az34 plants untreated with bacteria. In the latter case, RWC content was lowered to a greater extent (to 84%).

Stomatal conductance was decreased by NaCl addition was higher in Az34 than in Steptoe in both unstressed and salt-stressed plants and was not influenced by bacterial treatment (Figure 2B).

In the absence of stress, the rate of transpiration did not differ between Az34 and Steptoe, smaller leaf size of the former being obviously compensated by higher stomatal conductance. Transpiration was decreased by salt stress and increased by bacterial treatment (Figure 2C).

**Figure 3:**
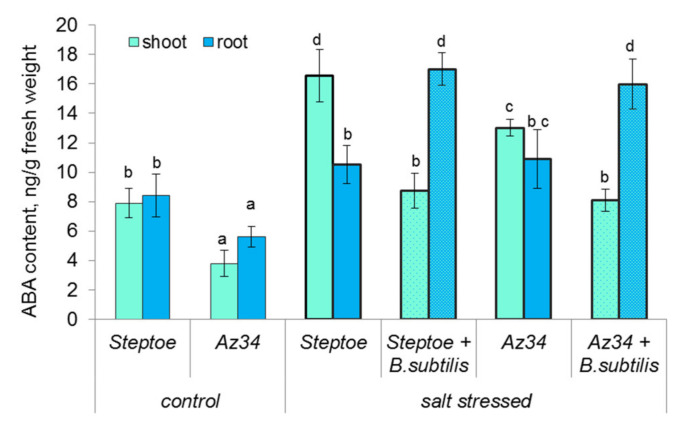
Influence of *B. subtilis* IB-22 on ABA content in shoots and roots of control (unstressed) and salt-stressed (100 mM NaCl) ABA-deficient barley mutant (Az34) and its parental cultivar Steptoe measured on the 8th day after bacterial treatment. Significantly different means are labeled with different letters at *p* ≤ 0.05, *n* = 6.

As expected, ABA concentration was lower both in roots and shoots of ABA-deficient mutant (Az34) than in its parental cultivar in control (unstressed) plants (Figure 3). Salt stress increased ABA content in the shoots of both genotypes. In roots, the content of this hormone was increased by 100 mM NaCl in Az34, while it remained the same in the roots of salt-stressed and unstressed Steptoe. Bacterial treatment increased the content of ABA in roots and decreased it in shoots of salt-stressed plants of both genotypes.

ABA concentration was about 20 ng mL^−1^ in bacterial culture media.

**Figure 4:**
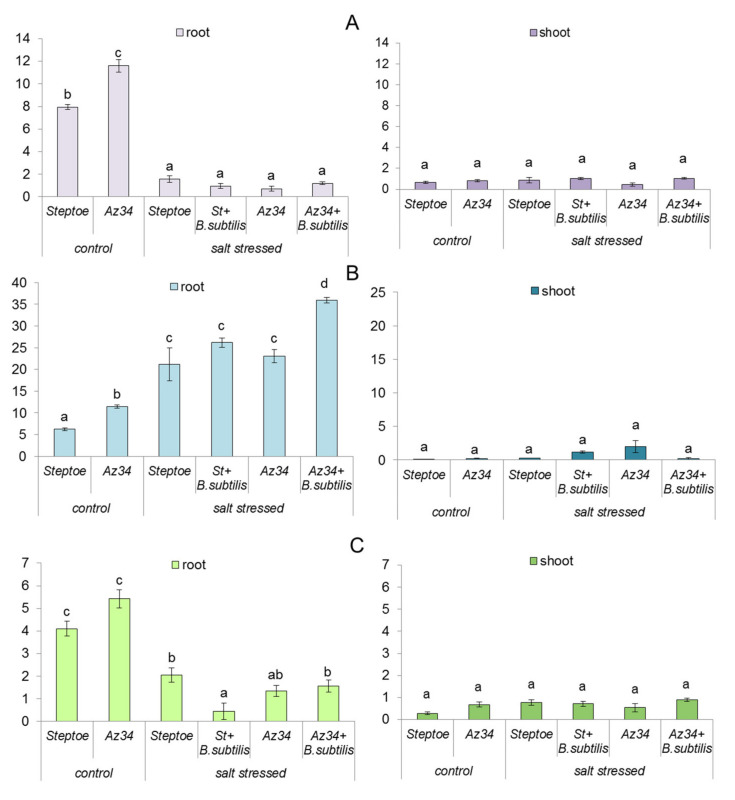
Effect of bacterial treatment of *B. subtilis* IB-22 on the content of transcripts of genes *HvNCED1* (**A**), *HvNCED2* (**B**), and *HvCYP707A1* (**C**) in roots and shoots of control (unstressed) and salt-stressed (100 mM NaCl) ABA-deficient barley mutant (Az34) and its parental cultivar Steptoe measured on the 7th day after bacterial treatment. The expression values of genes responsible for ABA metabolism in barley are normalized relative to the barley gene *HvGADPH*, encoding glyceraldehyde 3-phosphate dehydrogenase. Significantly different means for each gene are labeled with different letters (*n* = 6).

Expression of *HvNCED1* and *HvNCED2* genes encoding dioxygenase of 9-cis-epocorotinoids (key enzyme for ABA synthesis) was much greater in the roots than in the shoots of barley plants (Figure 4A,B).

In control (unstressed) plants, the abundance of root *HvNCED1* and *HvNCED2* transcripts was higher in Az34 than in Steptoe (Figure 4A,B). Root *HvNCED1* was down-regulated, while *HvNCED2* was up-regulated by salt stress (Figure 4B). The abundance of *HvNCED2* transcript was increased by bacterial treatment.

Expression of the *HvCYP707A1* gene-encoding hydroxylase involved in ABA catabolism was also mainly higher in the roots than in the shoots of barley plants (Figure 4C). The abundance of the transcripts of this gene in roots was decreased by the salt stress in the plants of both genotypes and by bacterial treatment of Steptoe.

## 3. Discussion

Growth-promoting effects of *Bacillus subtilis* IB22 have been shown by us previously on lettuce plants grown under normal water supply and drought [9] as well as on wheat plants grown under either laboratory [23,24] or field conditions [25]. In the present experiments, ABA-deficient barley mutant and its parental genotype have been used to address the importance of ABA for the growth-promoting effect of this bacterial strain. Decreased ABA concentration in Az34 plants detected in the present experiments is in accordance with the previous reports showing ABA deficiency in this mutant [26,27]. Low ABA concentration in these plants is due to mutation of the gene-encoding molybdenum cofactor of aldehyde oxidase catalyzing ABA synthesis from its aldehyde [28]. Despite the low activity of the corresponding enzyme resulting in lowered ABA concentration, this hormone was still present in the mutant plants (Figure 3). The presence of ABA in the mutants (although at a lower concentration than in the wild-type plants) was attributed to possible gene leakage [28] or the function of some genes with overlapping functions expressed at low levels [29]. ABA concentration in Az34 barley mutant was less decreased than in ABA-deficient tomato mutants [20]. This was possibly due to the high abundance of *HvNCED1* and *HvNCED2* transcripts in Az34 mutant compensating to some extent for low activity of oxidation of ABA aldehyde (Figure 4A,B). Still, despite the expression of the genes contributing to ABA synthesis in the mutant, the concentration of this hormone was lower in the mutant resulting in increased stomatal conductance (Figure 3 and Figure 2B), which could be related to the ability of this hormone to induce stomatal closure [30].

The smaller size of Az34 plants compared to Steptoe (Figure 1) may seem unexpected because ABA has historically been thought of as a growth inhibitor. Nevertheless, young tissues have high ABA levels, and some ABA-deficient mutant plants are severely stunted [14], suggesting that ABA is needed for normal growth. In the previous experiments, the ABA deficiency of Az34 was best manifested when a fast increase in ABA concentration was necessary and occurred in the wild-type plants (i.e., in response to air heating [27]).

ABA plays an important role in the control of water relations not only through limiting transpiration due to ABA-induced stomatal closure but also by increasing water flow brought about by ABA-induced increase in activity of water channels aquaporins [27,31]. Salt stress decreases the availability of water resulting in reduced mass and relative water content in barley plants (Figure 1A and Figure 2A) accompanied by stomatal closure (Figure 2B). It was important to relate these processes to the changes in ABA content in the stressed plants.

ABA concentration increased in salt-stressed Steptoe either in the shoots (in the absence of bacterial treatment) or in the roots (in bacteria-treated plants). Unlike previous short-term experiments [27], Az34 plants managed to accumulated ABA both in shoots and roots compared to unstressed Az34 plants after long-term salt stress (Figure 3). Accumulation of ABA in the salt-stressed plants of both genotypes was likely to be due to increased abundance of *HvNCED2* transcripts and decreased abundance of that of *HvCYP707A1* (Figure 4A,C), enabling accelerated ABA synthesis and reduced rate of its catabolism, correspondingly, in the stressed plants. Our data are in accordance with that obtained previously for barley plants showing expression of *HvNCED2* to be responsible for a significant increase in ABA level [22]. Nevertheless, ABA concentration in salt-stressed Az34 plants was lower than in salt-stressed Steptoe, which correlated with higher stomatal conductance and lower relative water content in the mutant. *HvNCED* gene was not likely to be involved in salt responses since its expression was decreased in roots of salt-stressed plants (Figure 4A). These data are in accordance with the report cited above [22], showing that the expression of the gene was variable and dependent on experimental conditions.

Introduction of bacteria into the rhizosphere increased ABA concentration in the roots of Az34 and Steptoe, which may be related to up-regulation of *HvNCED2* gene in Az34 roots and downregulation of *HvCYP707A1* in Steptoe roots compared to corresponding plants untreated with bacteria. The effects of bacterial treatment on the abundance of *HvNCED2* transcripts detected in the present experiments are partially in accordance with the reports showing that *Bacillus megaterium* up-regulation of *NCED* gene in tomato wild-type plants [20]. Alongside this, there is likely to be one more factor attributing to the increased concentration of ABA in the roots of bacteria-treated plants. The reason is that the bacterial strain used in the present work was capable of ABA synthesis. Although the concentration of ABA in the culture media of *Bacillus subtilis* IB-22 was about 10 times lower than reported for *Azospirillum brasilense* [15], it was still much higher than in *Bacillus aryabhattai* [32], while in the latter case, bacterial production of ABA was considered as a significant contribution to ABA accumulation in the treated soybean.

One more (and likely the most striking) feature of bacterial effect was in the opposite pattern of ABA distribution between roots and shoots compared to the salt-stressed plants untreated with bacteria. While ABA concentration was decreased in the shoots of salt-stressed plants by bacterial treatment, it was increased in the roots. A similar effect was observed previously in the salt-stressed wheat plants, where bacterial treatment resulted in the allocation of ABA to the roots [33]. Expression of *HvNCED1* and *HvNCED2* was much higher in the roots than in the shoots of plants (Figure 4A,B), indicating that the roots served as the main source of ABA in the barley plants of this age grown under indicated conditions. The coincidence of ABA accumulation in the roots of barley plants with the decline in its content in the shoots of bacteria-treated plants suggests inhibition of ABA transport from root to shoots by bacterial treatment. This suggestion is in accordance with the report showing that *Variovorax paradoxus* decreased xylem ABA flows from roots to shoots of *Pisum sativum* [34].

Accumulation of ABA in the roots accompanied by its decline in the shoots of wheat plants revealed previously under conditions of air heating resulted in increased root hydraulic conductance and water flow from the roots [35]. ABA-induced increase in root hydraulic conductance was related to an increase in the abundance of water channels (aquaporins) in the root epidermal cells [26]. Accumulation of ABA in the roots of bacteria-treated wheat plants was also accompanied by an increase in the root hydraulic conductivity [33]. Unlike Steptoe plants, where ABA content in the roots was rapidly increased by air heating accompanied by an increase in root hydraulic conductance, Az34 failed to either accumulate ABA or increase root hydraulic conductance [27]. It is likely that this barley mutant also failed to increase water flow to the shoots of salt-stressed plants in the present experiment resulting in lowered RWC (Figure 2A). Bacterial treatment increased ABA concentration in the roots of not only Steptoe but also of Az34, which was likely to maintain water flow resulting in the increase RWC in Az34 up to the level of Steptoe (Figure 2A and Figure 3). Although, unlike previous reports [9,33], *Bacillus subtilis* IB22 did not promote the root growth of barley plants in the present experiments, bacteria-induced increase in the root’s ability to supply water to shoots associated with an increase in ABA concentration in the roots contributed to the promotion of shoot growth. Accumulation of ABA in the roots of bacteria-treated plants was important for maintaining increased transpiration flow and enabling bacterial growth-promoting effect on barley plants.

Growth-promoting effect of *Bacillus subtilis* IB-22 on both ABA-deficient barley mutant and its parental cultivar revealed in the present experiments differ from the report showing that *Bacillus megaterium* caused growth inhibition in ABA-deficient tomato mutant [20]. This discrepancy may be due to the difference in either plant or bacteria species used in these experiments. ABA deficiency was compensated in barley mutant by the treatment with *Bacillus subtilis* IB-22 capable of producing this hormone, as shown in the present experiments. It is also important that in the present experiments, bacterial treatment was performed on stressed plants when the ability to accumulate ABA became most important. Present experiments demonstrate the importance of bacteria-induced ABA accumulation in the roots of salt-stressed barley plants for the growth-promoting effects of bacterial treatment. Further research is needed to confirm the importance of detected regularities at later stages of development.

## 4. Materials and Methods

Experiments were carried out on a spring barley (*Hordeum vulgare* L.) cultivar Steptoe (wild type) and its ABA-deficient mutant Az34 (a mutant generated in a Steptoe genetic background; both kindly provided by Prof. Ian Dodd at Lancaster University)

### 4.1. Experimental Design

Barley seeds were sterilized by soaking in a solution of 96% ethanol: 3% H_2_O_2_ (1:1, *v*/*v*) 5 min and then washed with distilled water multiple times. Then the seeds were stratified at 4 °C for 48 h and kept in the dark at room temperature for 24 h. Plants were grown on a light platform with a 14 h photoperiod, illumination of 400 μmol m^2^ s^−1^ of photosynthetically active radiation (PAR), and a temperature of 25/20 °C (day/night) in vessels with a volume of 500 cm^3^. To ensure drainage, a layer of gravel was placed at the bottom of the vessels.

After installing the glass tube for gas exchange, ten barley seedlings, whose coleoptile had a length of 0.3–0.5 cm, were planted in a vessel with sand preliminary sterilized by calcinations to prevent the introduction of undesirable bacteria and saturated. The sand in the pots was moistened with 50% Hoagland-Arnon solution [36] (containing 0.5 mM KNO_3_, 0.5 mM Ca(NO_3_)_2_, 0.1 mM KH_2_PO_4_, 0.1 mM MgSO_4,_ and essential microelements) to 90% of the total moisture capacity. Sand in half of the pots was moistened with 100 mM NaCl in 50% Hoagland-Arnon solution. The electrical conductivity of sand moistened with NaCl solution was about 10 dS/m and did not significantly decrease during the course of the experiment; therefore, NaCl was given only once. The bacterial suspension (1 mL (10^8^ CFU mL^−1^) per seedling) was added simultaneously with planting to the rhizosphere by applying to the surface of the soil around the roots of each seedling. Bacteria were grown as described below. The sand moisture was maintained at 80% of the total moisture capacity by watering the plants with distilled water. The amount of water required for irrigation was calculated by weighing vessels with plants daily. To maintain nutrition, the plants were watered every other day with 10 mL of 10% Hoagland-Arnon solution/vessel.

Weights of shoots and roots and length of leaves were measured on the 8th day after bacterial treatment.

### 4.2. Bacterial Strain and Culture Media

Gram-positive aerobic cytokinin-producing bacterium *Bacillus subtilis* IB-22 (GenBank MT590663) [23] from the collection of microorganisms of Ufa Institute of Biology, RAS, were used for bacterial treatment. Inoculates for bacterial treatments were obtained by cultivating *B. subtilis* IB-22 on K1 medium as described [34]. The strain of microorganisms was cultured in Erlenmeyer flasks on a shaker (160 rpm) for 72 h at 37 °C, and bacterial biomass was applied to the rhizosphere as described above. The concentration of ABA in bacterial culture media was measured 5 days after the start of cultivation.

### 4.3. ABA Assay

For the hormone assay, shoots and roots of 4 plants were sampled per replicate (*n* = 6) from different pots on the 8th day after bacterial treatment. A total of 1 mL of culture media was sampled on the 5th day of bacterial cultivation. The hormones were extracted from plants for 16 h with 80% ethanol in a ratio of 1:10. Then the alcohol extract separated by filtration was evaporated to an aqueous residue. ABA from the aqueous residue of plant extract or bacterial culture media was extracted with diethyl ether as described [37]. In short, ABA was partitioned with diethyl ether from the aqueous residue after its dilution with distilled water and acidification with HCl to pH 2.5 (ratio of organic to aqueous phases being 1:3). Then, ABA was transferred from the organic phase into a solution of NaHCO_3_ (ratio of aqueous/organic phase being 1:3), reextracted from the acidified aqueous phase with diethyl ether, ABA quantitative determination was performed with ELISA using specific antibodies as described [26,34]. The reliability of the method was due to the specificity of the antibodies obtained against ABA and the use of a modified ABA extraction method based on reducing the volume of extractants at each stage of extraction and re-extraction, which allows efficient extraction of ABA while reducing the amount of extracted impurities. The sufficiency of ABA purification prior to immunoassay was proved by studying the chromatographic distribution of the immunoreactive material, which showed that the peak of immunoreactivity coincides only with the position of the internal ABA standard.

### 4.4. Total Barley RNA

Total barley RNA was extracted from 100 mg of roots and shoots (one plant of average size for each of 3 replicates) on the seventh day after bacterial treatment, using TRIzol™ Reagent (Sigma, Germany) according to the manufacturer’s instructions. The potential contaminating DNA was digested with DNaseI (Synthol, Russia). First-strand cDNA was synthesized using the M−MLV reverse transcriptase (Fermentas). Oligo(dT)15 was used as a primer, and the reverse transcription reagents were incubated at 37 °C for 1 h in a total volume of 25 μL. After ten-fold dilution, 2 μL of the synthesized cDNA was used for quantitative real-time polymerase chain reaction (qPCR). The primers for qPCR (Table 1) were designed based on the cDNA sequence using PrimerQuest™ Tool.

Quantitative PCR was performed by polymerase chain reaction in real time using a set of predefined reagents EvaGreenI (Synthol, Russia) and CFX Connect real-time PCR Detection System device (BioRad Laboratories, Hercules, CA, USA). The qPCR program was as follows: 95 °C for 5 min; 40 cycles of 95 °C for 15 s and at 60 °C for 20 s and 72 °C 30 s. After the final PCR cycle, a melting curve analysis was conducted to determine the specificity of the reaction (at 95 °C for 15 s, 60 °C for 1 min, and 95 °C for 15 s). The efficiency of each primer pair was determined using 10-fold cDNA dilution series in order to reliably determine the fold changes. To standardize the data, barley gene *HvGADPH_2* (GenBank Accession No. EF409606) was used as an internal reference for the real-time qPCR analysis. The quantification of gene expression was performed using CFX Connect Real-time PCR Detection System (BioRad Laboratories, Hercules, CA, USA). All reactions, including the nontemplate control, were performed three times. The threshold values (CT) generated from the CFX Connect real-time PCR Detection System software tool (Applied Biosystems) were employed to quantify the relative gene expression using the comparative threshold (delta CT) method. Three independent biological replicates were performed for each experimental variant.

### 4.5. Water Relation Measurements

#### 4.5.1. Transpiration

*Transpiration* was measured by the weight loss of pots with 8-day-old plants, in which soil was covered for 4 h with parafilm to prevent water evaporation.

#### 4.5.2. Relative Water Content (RWC)

To determine relative water content (RWC), on the 8th day after bacterial treatment, mature first leaves of four plants were weighed and immersed in distilled water with the base; a vessel was tightly closed to saturate the air with moisture and placed in darkness at room temperature. After 24 h, the turgid weight (TW) was determined after blotting, and the dry mass was determined after drying for 24 h at 80 °C. Fresh weight (FW), dry weight (DW), and TW were used to determine relative water content: RWC = (FW − DW)/(TW − DW).

### 4.6. Stomatal Conductance

Stomatal conductance was measured with a porometer (MKDeltaT Decices, United Kingdom) on the 8th day after bacterial treatment.

### 4.7. Statistics

The data were processed using the Statistica version 10 software (Statsoft, Moscow, Russia). In figures and tables, data are presented as mean ± standard error. The significance of differences was assessed by ANOVA followed by Duncan’s test (*p* ≤ 0.05).

## Figures and Tables

**Table 1:** Sequences of primers used for qRT-PCR (annealing temperature 60 °C).

Genes	Strand	5′ to 3′ Primer Sequences	GenBank Accession Number
*HvNCED1*	Forward	CCCCTATGGCTTCCACGGCACATT	AB239297
	Reverse	GTGATGAGTAACCGCCGCTAACTG	
*HvNCED2*	Forward	CGGGTACATTCTCACCTTCGTGCA	AB239298
	Reverse	CTGGCAACTTCCTCTTTCCATGTCC	
*HvCYP707A1*	Forward	CCACCAAGTACAGATGGTCTAC	AB239299
	Reverse	TCCGAAGGAGGAAGACATAGA	
*HvGADPH*	Forward	GCCACTATTCTTCAGGGACTT	EF409626
	Reverse	CTTCTTGGCACCACCCTAATA	

## Data Availability

The data presented in this study are available in the graphs and tables provided in the manuscript.

## References

[1] Dodd I.C., Perez-Alfocea F. (2012). Microbial amelioration of crop salinity stress. J. Exp. Bot..

[2] Egamberdieva D., Wirth S., Bellingrath-Kimura S.D., Mishra J., Arora N.K. (2019). Salt-Tolerant Plant Growth Promoting Rhizobacteria for Enhancing Crop Productivity of Saline Soils. Front. Microbiol..

[3] Mokrani S., Nabti E., Cruz C. (2020). Current advances in plant growth promoting bacteria alleviating salt stress for sustainable agriculture. Appl. Sci..

[4] He A.-L., Niu S.-Q., Zhao Q., Li Y.-S., Gou J.-Y., Gao H.-J., Suo S.-Z., Zhang J.-L. (2018). Induced Salt Tolerance of Perennial Ryegrass by a Novel Bacterium Strain from the Rhizosphere of a Desert Shrub Haloxylon ammodendron. Int. J. Mol. Sci..

[5] Ruzzia M., Aroca R. (2015). Plant growth-promoting rhizobacteria act as biostimulants in horticulture. Sci. Hort..

[6] Shi T.-Q., Peng H., Zeng S.-Y., Ji R.-Y., Shi K., Huang H., Ji X.-J. (2017). Microbial production of plant hormones: Opportunities and challenges. Bioengineered.

[7] Backer R., Rokem J.S., Ilangumaran G., Lamont J., Praslickova D., Ricci E., Subramanian S., Smith D.L. (2018). Plant Growth-Promoting Rhizobacteria: Context, Mechanisms of Action, and Roadmap to Commercialization of Biostimulants for Sustainable Agriculture. Front. Plant Sci..

[8] Kudoyarova G., Arkhipova T., Korshunova T., Bakaeva M., Loginov O., Dodd I.C. (2019). Phytohormone mediation of interactions between plants and non-symbiotic growth promoting bacteria under edaphic stresses. Front. Plant Sci..

[9] Arkhipova T.N., Prinsen E., Veselov S.U., Martinenko E.V., Melentiev A.I., Kudoyarova G.R. (2007). Cytokinin producing bacteria enhance plant growth in drying soil. Plant Soil.

[10] Liu F., Xing S., Ma H., Du Z., Ma B. (2013). Cytokinin-producing, plant growth-promoting rhizobacteria that confer resistance to drought stress in *Platycladus orientalis* container seedlings. Appl Microbiol Biotechnol..

[11] Poupin M.J., Greve M., Carmona V., Pinedo I. (2016). A complex molecular interplay of auxin and ethylene signaling pathways is involved in arabidopsis growth promotion by *Burkholderia phytofirmans* PsJN. Front. Plant Sci..

[12] Agarwal P., Singh P.C., Chaudhry V., Shirke P.A., Chakrabarty D., Farooqui A., Nautiya C.S., Sane A.P., Sane V.A. (2019). PGPR-induced OsASR6 improves plant growth and yield by altering root auxin sensitivity and the xylem structure in transgenic *Arabidopsis thaliana*. J. Plant Physiol..

[13] Duca D.R., Glick B.R. (2020). Indole-3-acetic acid biosynthesis and its regulation in plant-associated bacteria. Appl. Microbiol. Biotechnol..

[14] Finkelstein R. (2013). Abscisic Acid Synthesis and Response. Arabidopsis Book.

[15] Cohen A.C., Bottini R., Piccoli P.N. (2008). *Azospirillum brasilense* Sp 245 produces ABA in chemically-defined culture medium and increases ABA content in arabidopsis plants. Plant Growth Regul..

[16] Yuzikhin O.S., Gogoleva N.E., Shaposhnikov A.I., Konnova T.A., Osipova E.V., Syrova D.S., Ermakova E.A., Shevchenko V.P., Nagaev I.Y., Shevchenko K.V. (2021). Rhizosphere bacterium rhodococcus sp. P1Y metabolizes abscisic acid to form dehydrovomifoliol. Biomolecules.

[17] Belimov A.A., Dodd I.C., Safronova V.I., Dumova V.A., Shaposhnikov A.I., Ladatko A.G., Davies W.J. (2014). Abscisic acid metabolizing rhizobacteria decrease ABA concentrations *in planta* and alter plant growth. Plant Physiol. Biochem..

[18] Kang S.M., Khana A.L., Waqasa M., You Y.H., Kim J.H., Kim J.G., Hamayun M. (2014). Plant growth-promoting rhizobacteria reduce adverse effects of salinity and osmotic stress by regulating phytohormones and antioxidants in *Cucumis sativus*. J. Plant Interact..

[19] Chen L., Liu Y., Wu G., Njeri K.V., Shen Q., Zhang N., Zhang R. (2016). Induced maize salt tolerance by rhizosphere inoculation of *Bacillus amyloliquefaciens* SQR9. Physiol Plant..

[20] Porcel R., Zamarreno A.M., Garcia-Mina J.M., Aroca R. (2014). Involvement of plant endogenous ABA in Bacillus megaterium PGPR activity in tomato plants. BMC Plant Biol..

[21] Bharti N., Pandey S.S., Barnawal D., Patel V.K., Kalra A. (2016). Plant growth promoting rhizobacteria *Dietzia natronolimnaea* modulates the expression of stress responsive genes providing protection of wheat from salinity stress. Sci. Rep..

[22] Chono M., Honda I., Shinoda S., Kushiro T., Kamiya Y., Nambara E., Kawakami N., Kaneko S., Watanabe Y. (2006). Field studies on the regulation of abscisic acid content and germinability during grain development of barley: Molecular and chemical analysis of pre-harvest sprouting. J. Exp. Bot..

[23] Kudoyarova G.R., Melentiev A.I., Martynenko E.V., Arkhipova T.N., Shendel G.V., Kuzmina L.Y., Dodd I.C., Veselov S.Y. (2014). Cytokinin producing bacteria stimulate amino acid deposition by wheat roots. Plant Physiol. Biochem..

[24] Arkhipova T.N., Evseeva N.V., Tkachenko O.V., Burygin G.L., Vysotskaya L.B., Akhtyamova Z.A., Kudoyarova G.R. (2020). Effect of rhizobacteria on phytohormone status of potato microclones under osmotic stress *in vitro*. Biomolecules.

[25] Wilkinson S., Kudoyarova G.R., Veselov D.S., Arkhipova T.N., Davies W.J. (2012). Plant hormone interactions: Innovative targets for crop breeding and management. J. Exp. Bot..

[26] Sharipova G., Veselov D., Fricke W., Dodd I., Katsuhara M., Furuichi T., Ivanov I., Veselov S. (2016). Exogenous application of abscisic acid (ABA) increases root and cell hydraulic conductivity and abundance of some aquaporin isoforms in the ABA deficient barley mutant Az34. Ann. Bot..

[27] Veselov D.S., Sharipova G.V., Veselov S.Y., Dodd I.C., Ivanov I., Kudoyarova G.R. (2018). Rapid changes in root *HvPIP2;2* aquaporins abundance and ABA concentration are required to enhance root hydraulic conductivity and maintain leaf water potential in response to increased evaporative demand. Funct. Plant Biol..

[28] Walker-Simmons M., Kudrna D.A., Warner R.L. (1989). Reduced accumulation of aba during water stress in a molybdenum cofactor mutant of barley. Plant Physiol..

[29] Tan B.C., Schwartz S.H., Zeevaart J.A., McCarty D.R. (1997). Genetic control of abscisic acid biosynthesis in maize. Proc. Natl. Acad. Sci. USA.

[30] Davies W.J., Kudoyarova G., Hartung W. (2005). Long-distance ABA signaling and its relation to other signaling pathways in the detection of soil drying and the mediation of the plant’s response to drought. J. Plant Growth Regul..

[31] Vandeleur R.K., Sullivan W., Athman A., Jordans C., Gilliham M., Kaiser B.N., Tyerman S.D. (2014). Rapid shoot-to-root signalling regulates root hydraulic conductance via aquaporins. Plant Cell Environ..

[32] Park Y.-G., Mun B.-G., Kang S.-M., Hussain A., Shahzad R., Seo C.-W., Kim A.-Y., Lee S.-U., Oh K.Y., Lee D.Y. (2017). *Bacillus aryabhattai* SRB02 tolerates oxidative and nitrosative stress and promotes the growth of soybean by modulating the production of phytohormones. PLoS ONE.

[33] Arkhipova T., Martynenko E., Sharipova G., Kuzmina L., Ivanov I., Garipova M., Kudoyarova G. (2020). Effects of plant growth promoting rhizobacteria on the content of abscisic acid and salt resistance of wheat plants. Plants.

[34] Jiang F., Chen L., Belimov A.A., Shaposhnikov A.I., Gong F., Meng X., Hartung W., Jeschke D.W., Davies W.J., Dodd I.C. (2012). Multiple impacts of the plant growth-promoting rhizobacterium *Variovorax paradoxus* 5C-2 on nutrient and ABA relations of *Pisum sativum*. J. Exp. Bot..

[35] Kudoyarova G., Veselova S., Hartung W., Farhutdinov R., Veselov D., Sharipova G. (2011). Involvement of root ABA and hydraulic conductance in the control of water relations in wheat plants exposed to increased evaporative demand. Planta.

[36] Hoagland D.R., Arnon D.I. (1950). The Water-Culture Method for Growing Plants Without Soil.

[37] Veselov D.S., Sharipova G.V., Veselov S.U., Kudoyarova G.R. (2008). The effects of NaCl treatment on water relations, growth and ABA content in barley cultivars differing in drought tolerance. J. Plant Growth Regul..

